# New Dental Depot

**Published:** 1862-02

**Authors:** 


### NEW DENTAL DEPOT.

We take pleasure in announcing the establishment of a new Dental Depot in this city, by that true and long-tried friend of the profession, Mr. James Leslie, who has won an enviable reputation as a manufacturer of superior gold foil. He now in connection with his foil furnishes all other dental goods, and of a superior quality, and at prices to which none can object. His stock is all new, and his teeth especially constitute as fine an assortment as we have ever seen.

He keeps all dental goods that are in demand; his goods were bought for cash, and will be sold on the same terms, and we know that those who deal with him will not have cause of complaint.

His Depot is on the north-west corner of Fourth and Race sts., where he will be happy at all times to meet the members of the profession. T.

